# ADP and Thromboxane Inhibitors Both Reduce Global Contraction of Clot Length, While Thromboxane Inhibition Attenuates Internal Aggregate Contraction

**DOI:** 10.1055/a-1832-9293

**Published:** 2022-06-13

**Authors:** Kevin T. Trigani, Michael E. DeCortin, Scott L. Diamond

**Affiliations:** 1 Department of Chemical and Biomolecular Engineering, Institute for Medicine and Engineering, University of Pennsylvania, Philadelphia, Pennsylvania, United States

**Keywords:** platelet, contraction, ADP, thromboxane A2, thrombosis

## Abstract

Platelet contractility drives clot contraction to enhance clot density and stability. Clot contraction is typically studied under static conditions, with fewer studies of wall-adherent platelet clots formed under flow. We tested the effect of inhibitors of ADP and/or thromboxane A2 (TXA2) signaling on clot contraction. Using an eight-channel microfluidic device, we perfused PPACK-treated whole blood (WB) ± acetylsalicylic acid (ASA), 2-methylthioAMP (2-MeSAMP), and/or MRS-2179 over collagen (100/s) for 7.5 min, then stopped flow to observe contraction for 7.5 minutes. Two automated imaging methods scored fluorescent platelet percent contraction over the no-flow observation period: (1) “global” measurement of clot length and (2) “local” changes in surface area coverage of the numerous platelet aggregates within the clot. Total platelet fluorescence intensity (FI) decreased with concomitant decrease in global aggregate contraction when ASA, 2-MeSAMP, and/or MRS-2179 were present. Total platelet FI and global aggregate contraction were highly correlated ( *R* ^2^ = 0.87). In contrast, local aggregate contraction was more pronounced than global aggregate contraction across all inhibition conditions. However, ASA significantly reduced local aggregate contraction relative to conditions without TXA2 inhibition. P-selectin display was significantly reduced by ADP and TXA2 inhibition, but there was limited detection of global or local aggregate contraction in P-selectin-positive platelets across all conditions, as expected for densely packed “core” platelets. Our results demonstrate that global aggregate contraction is inhibited by ASA, 2-MeSAMP, and MRS-2179, while ASA more potently inhibited local aggregate contraction. These results help resolve how different platelet antagonists affect global and local clot structure and function.

## Introduction

Clot contraction is an important process that occurs in hemostasis to ensure clot resolution and return of unobstructed blood flow. Contraction involves a number of elements, including fibrin, fibrinogen, platelets, red blood cells (RBCs), von Willebrand's factor (VWF), and collagen, among others. The process of clot contraction actually consists of a number of distinct processes that occur including, but not limited to, reduction of clot size, 1 2 3 4 stiffening of the clot matrix, 1 5 6 deformation of RBCs, 7 8 extrusion of procoagulant platelets, 9 10 and redistribution of fibrin. 1 4 The significance of contraction as a process can be seen in diseases where there are defects in nonmuscle myosin, resulting in increased bleeding. 11 Additionally, reduced contraction can lead to increased risk of venous thromboembolism 12 13 which could ultimately lead to acute ischemic stroke. 14

Platelet contraction via cytoskeletal actin and myosin plays a key role in strengthening and stabilizing clots. 11 15 16 Nonmuscle myosin is a driving force of platelets' contractile ability; when blebbistatin is present to inhibit nonmuscle myosin, clot contraction is significantly reduced. 11 16 17 Although platelets contribute significantly to clot contraction, many viscoelastic measurement techniques to evaluate contraction require the presence of thrombin and/or fibrin which does not allow for the independent evaluation of the contractile ability of platelets. 1 18 19 20 Additionally, many of these contraction measurement techniques are not performed under physiologic or near-physiologic conditions. Our in vitro microfluidic system allows us the ability to observe clot development, including contraction on a macro scale, evaluating multiple platelet aggregates in a fully formed clot in a physiologically relevant environment.

**Visual Summary:**
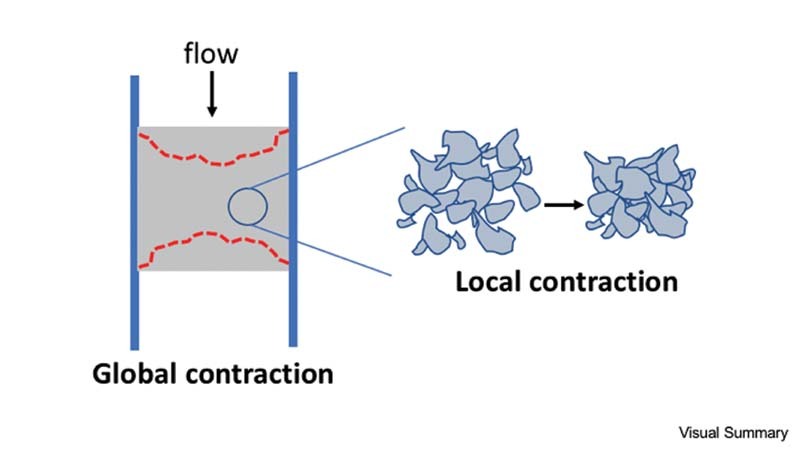
We developed a microfluidic assay to evaluate clot contraction in the presence of ADP and/or thromboxane (TX) A2 inhibitors. Clot contraction was measured using two distinct, automated metrics to measure contraction: a global contraction measurement and local contraction measurement.

On binding of collagen and VWF, platelets release ADP and thromboxane A2 (TXA2) which propagates further activation of nearby platelets to contribute to the stability of a growing platelet mass. 6 21 22 As shown in the core/shell model, Stalker et al suggest that ADP and TXA2 moderate the growth of the P-selectin(-) shell region of the clot, while ADP and TXA2 inhibitors have a limited effect on the stable core region, making these inhibitors ideal for limiting platelet accumulation while also preventing bleeding. 23 As such, TXA2 inhibitors, like aspirin, and ADP inhibitors, like clopidogrel, are commonly used in a number of patient conditions, but the effects of these types of these drugs on clot contraction is not fully elucidated. Ting et al evaluated ADP and TXA2 inhibitors on platelet contractile forces using a microfluidic device with a block and post. They observed that acetylsalicylic acid (ASA, or asiprin), a COX-1 inhibitor that prevents TXA2 production, and 2-methylthioadenosine 5'-monophosphate (2-MeSAMP) a P _2_ Y _12_ inhibitor which prevents ADP from binding and propagating, significantly reduce platelet forces and aggregate size. However, these experiments were performed at 8,000/s which is at the very high end of arterial flow shear rates. 24 Additionally, the block and post device to measure platelet contractile force has limited physiological relevance.

Here, we wanted to evaluate contraction in our microfluidic assay that closely mimics in vivo dynamics of a growing clot. We also evaluated three platelet antagonists, that is, ASA, 2-MeSAMP, and MRS-2179, which is a P _2_ Y _1_ inhibitor that prevent ADP from binding and propagating. We added these platelet antagonists to PPACK-treated WB to ensure thrombin and fibrin inhibition. This allowed us to focus primarily on platelet-platelet interactions and not platelet-fibrin interactions. We wanted to differentiate contraction of platelet aggregates in both the core and shell regions, utilizing CD61 and P-selectin antibodies, to measure aggregate contraction in different regions of the clot. Additionally, we measured contraction using two different algorithms that we developed, one for global contraction, measuring clot contraction on a macro scale, and looking at fully formed clots with multiple platelet aggregates; and one for local contraction, measuring clot contraction on a micro scale, and looking at how individual platelet aggregates contract within a larger clot ( Visual Summery ). We found that both ADP and TXA2 inhibitors limited global aggregate contraction and had an additive effect when used in combination. However, TXA2 inhibition had a significant effect on reducing local aggregate contraction, while ADP inhibition did not. Lastly, P-selectin + platelets demonstrated limited global and local aggregate contraction, both in the presence and absence of any ADP or TXA2 inhibition. Taken together, the results of this study suggest that ADP and TXA2 inhibition may limit aggregate contraction on a macro scale, and TXA2 inhibition, in particular, may limit aggregate contraction on a microscale which has fundamental repercussions in terms of clot development and clot resolution.

## Methods

### Reagents and Materials

Reagents were prepared and kept at appropriate conditions prior to use in experiments: Phe-Pro-Arg-chloromethylketone (PPACK; EMD Millipore, Burlington, Massachusetts, United States), acetylsalicylic acid (ASA; Sigma-Aldrich, St. Louis, Missouri, United States), 2-methylthioadenosine 5'-monophosphate (2-MeSAMP; Sigma-Aldrich, St. Louis, Missouri, United States), MRS-2179 (Tocris, Minneapolis, Minnesota, United States), AF647 anti-human CD62P (P-Selectin) Antibody (BioLegend, San Diego, California, United States), AF488 mouse anti-human CD61 (Bio-Rad Laboratories, Hercules, California, United States), Sigmacote (Sigma-Aldrich, St. Louis, Missouri, United States), and type-I fibrillar collagen (Chrono-log, Havertown, Pennsylvania, United States).

### Preparation of Microfluidic Device with Collagen Surface

Glass slides were rinsed with ethanol and dried with filtered air. Slides were then rinsed with Sigmacote and then deionized water to create a hydrophobic surface. A patterning device was vacuum sealed to the glass slide. Fibrillar collagen (5 µL) was perfused through the patterning channel (250 µm wide × 60 µm high) of the patterning device to create a 250-μm wide strip of collagen for all experiments, as previously described. 25 26 27 28 Finally, collagen was rinsed with 20-μL 0.5% bovine serum albumin (BSA) buffer, and the patterning device was then removed from the glass slide, leaving a strip of collagen.

### Blood Collection and Preparation

Blood donors were self-reported as medication free for at least 7 days prior to blood collection. All donors provided informed consent under approval of the University of Pennsylvania Institutional Review Board. Blood was obtained via venipuncture into a syringe containing PPACK (1:100 v/v; final concentration of 100 µmol/L). P-selectin and anti-CD61 fluorophores were then added to WB, both in a 1:50 v/v dilution. ADP and TXA2 inhibitors were added where needed, resulting in the following final concentrations: ASA (100 µM), 2-MeSAMP (100 µM), and MRS-2179 (10 µM).

### Eight-Channel Microfluidic Device Contraction Assay

After collagen was patterned, an eight-channel microfluidic device was placed over the collagen strip and vacuum sealed with channels perpendicular to the collagen strip. Thus, each channel contained a 250 µm × 250 µm collagen patch. Each channel had a height of 120 µm. Then 35 µL of 0.5% BSA was added to each of the eight wells and perfused through each channel. After blood was drawn, fluorophores were added, followed by ADP or TXA2 inhibitors (ASA, 2-MeSAMP, and/or MRS-2179) or their corresponding controls. ASA stock was dissolved in 10% DMSO with a final concentration of 0.1% DMSO in blood (1:100 ASA dilution). The control for ASA was a 10% DMSO solution (1:100 dilution; 0.1% DMSO final concentration in blood). The 2-MeSAMP and MRS-2179 were both dissolved in water; the control for 2-MeSAMP and MRS-2179 was HEPES buffered saline (HBS). Once agonists or controls were added to blood, blood of varying conditions was loaded into the corresponding wells. Blood was perfused through channels by a 1,000-µL syringe (Hamilton Co., Reno, Nevada, United States) with a fixed rate to induce blood flow. The syringe pump (Harvard PHD 2000; Harvard Apparatus, Holliston, Massachusetts, United States) was set to 24 µL/min, resulting in an initial venous shear rate of 100/s for all experiments. Blood was perfused for 7.5 minutes, and then flow was paused (0/s) at 7.5 minutes, for the remaining 7.5 minutes of the experiment, for a total experiment time of 15 minutes. The epifluorescence microscope (Olympus I × 81; Olympus America Inc, Central Valley, Pennsylvania, United States) captured individual time frames at chosen locations with chosen wavelengths.

### Image Analysis

#### Fluorescence Measurements

Fluorescence intensity (FI) of fluorophores (CD61 and P-selectin) was measured over time, with images of each clot uploaded and compiled into a time sequence in ImageJ. The initial image ( *t* = 0 seconds) was subtracted from subsequent images to subtract for any background fluorescence. Fluorescence was measured using a rectangular subsection of the central two-thirds of the clot area, to avoid any edge effects. FI values were exported to Matlab where plots were made of the FI values and experiment time. Values in plots are average ± standard deviation of FI values over time.

#### Global Contraction Measurements

Images of CD61 and P-selectin fluorescence were uploaded to ImageJ. We wanted to observe the extent of contraction in clots on the collagen surface, which was 250 µm × 250 µm. However, in many instances, platelets aggregated above and below the collagen surface in the direction of flow. To include this in our contraction measurements, images of individual clots were made to be 250 µm × 300 µm, with the 300-µm dimension in the direction of flow, incorporating all platelets in the clot above and below the collagen strip. Images were then uploaded to Matlab and binarized to allow for automated contraction measurements. The threshold for binarizing images was chosen to be half of the average fluorescence of the image ( Supplementary Fig. S1 ). Binary images had platelets represented in white and empty space in black.

Once images were binary in Matlab, we measured global contraction by building a Matlab algorithm that automatically drew five lines through the central portion of the clot in the same direction as flow. One line was drawn at the middle (of the x direction) pixel of the clot. Four additional lines were drawn at the following locations, relative to the middle line: −50 pixels, −25 pixels, +25 pixels, and +50 pixels (each in the x direction). Each line was drawn from the top most platelet (white) pixel to the bottom most platelet (white) pixel (in the y direction). This was done for images at 7.5 and 15 minutes of the same clot. The lengths of the five lines at 7.5 minutes were averaged, as were the lengths of the five lines at 15 minutes. The average lengths at 7.5 and 15 minutes were then used to calculate the percent global contraction ( Supplementary Fig. S2 ). This was done for both CD61 and P-selectin images.

#### Local Contraction Measurements

In addition to global contraction, we used binary images in Matlab to calculate local contraction for CD61 and P-selectin images. Binary images at 7.5 and 15 minutes for a particular clot were measured in Matlab for their percent platelet coverage or percent of the image containing white space (representing platelets). The percent coverage between 7.5 and 15 minutes images were each used to calculate local contraction ( Supplementary Fig. S3 ). This was done for both CD61 and P-selectin images.

### Statistical Analyses

Statistical differences between conditions were calculated using unpaired Welch's *t* -tests in GraphPad (ns signifies *p* > 0.05).

## Results

### Acetylsalicylic Acid, 2-MeSAMP, and MRS-2179 Have an Additive Effect on Limiting Platelet Deposition.

**Fig. 1:**
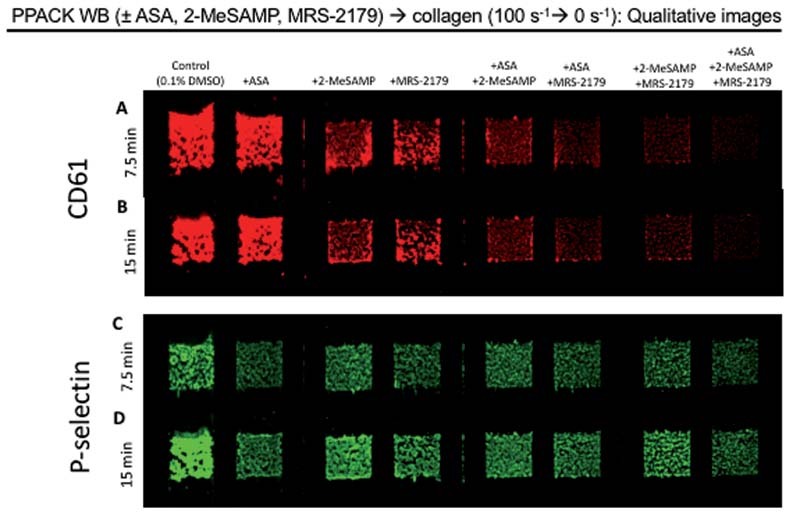
ASA, 2-methylthio (Me)-SAMP, and MRS-2179 have an additive effect on limiting platelet deposition, but do not have an additive effect on limiting P-selectin display. PPACK WB with and without ASA, 2-MeSAMP, and MRS-2179 was perfused over collagen at 100/s for 7.5 minutes, then flow was stopped for 7.5 minutes. CD61 fluorophore was added to label platelets, with images taken at 7.5 minutes **(A)** and 15 minutes **(B)** . P-selectin fluorophore was added to label platelet activation via alpha-granule release, with images taken at 7.5 minutes **(C)** and 15 minutes **(D)** . ASA, acetylsalicylic acid; WB, whole blood.

**Fig. 2:**
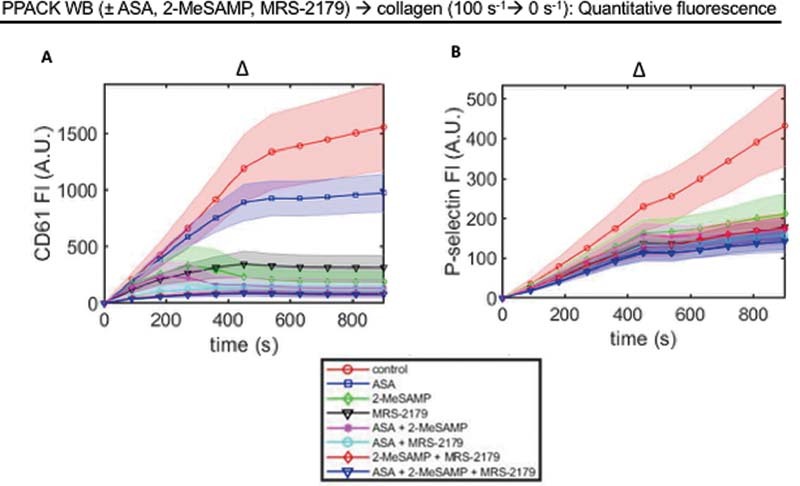
Quantitative fluorescence data from Fig. 1 . PPACK WB with and without ASA, 2-methylthio (Me)-SAMP, and MRS-2179 was perfused over collagen at 100/s for 7.5 minutes, then flow was stopped for 7.5 minutes. Platelet **(A)** and P-selectin **(B)** fluorescence intensities were measured throughout the course of the experiment amongst the different conditions. Delta signifies change from 100 to 0/s flow rate at 7.5 minutes. (N = 3 donors, *n* = 6 clots for each panel; AU = arbitrary units). ASA, acetylsalicylic acid; WB, whole blood.

To measure how ADP and TXA2 inhibition affect clot contraction, we perfused PPACK WB with and without ASA, 2-MeSAMP, and MRS-2179 over collagen at 100/s for 7.5 minutes, followed by stopping flow for 7.5 minutes. ASA, 2-MeSAMP, and MRS-2179 were each added individually, and in combination with each other. We added CD61 fluorophore to WB to label for platelets. By 7.5 minutes, platelets had deposited normally in control conditions, while there appeared to be slightly reduced platelet deposition in +ASA conditions, and considerably reduced platelet deposition in +2-MeSAMP, +MRS-2179, and all combinatorial conditions ( Figs. 1A , 2A ). From 7.5 to 15 minutes, with flow stopped, platelet FI in control continued to increase slightly, but at a slower rate than from 0 to 7.5 minutes. This increase in platelet FI while flow had stopped was likely due from contraction. All other conditions remained nearly constant from 7.5 to 15 minutes ( Figs. 1B , 2A ). Notably, the combinatorial conditions (with 2 or 3 ADP and/or TXA2 inhibitors present) had lower final FI than either of the conditions with only one inhibitor present, suggesting an additive effect of ASA, 2-MeSAMP, and MRS-2179 on reducing platelet deposition.

### Acetylsalicylic Acid, 2-MeSAMP, and MRS-2179 Did Not Have an Additive Effect on Limiting P-Selectin Display.

In addition to CD61, we also added P-selectin fluorophore to WB to label for alpha-granule release. By 7.5 minutes, P-selectin display was prominent in each condition, with the control showing slightly greater fluorescence than the conditions with ADP/TXA2 inhibitors present ( Figs. 1C and 2B ). From 7.5 to 15 minutes, with flow stopped, P-selectin fluorescence continued to increase steadily in the control condition ( Figs. 1D and 2B ). This continued increase was greater than the increase in platelet deposition from 7.5 to 15 minutes, indicating that the increase in P-selectin from 7.5 to 15 minutes was likely not solely due from contraction. Additionally, P-selectin display in conditions with ADP and/or TXA2 inhibitors continued to slightly increase from 7.5 to 15 minutes which was not the case for platelet deposition. Like platelet deposition, P-selectin display appeared to be markedly reduced by the presence of any ADP or TXA2 inhibition. However, unlike with platelet deposition, ADP and/or TXA2 inhibition did not appear to have a strong additive effect on reducing P-selectin fluorescence, as differences in final fluorescence among different conditions with ADP and/or TXA2 inhibitors showed fairly similar FI values, with a few, minimal statistical differences between different conditions.

### Acetylsalicylic Acid, 2-MeSAMP, and MRS-2179 Have a Limiting Effect on Global Aggregate Contraction

**Fig. 3:**
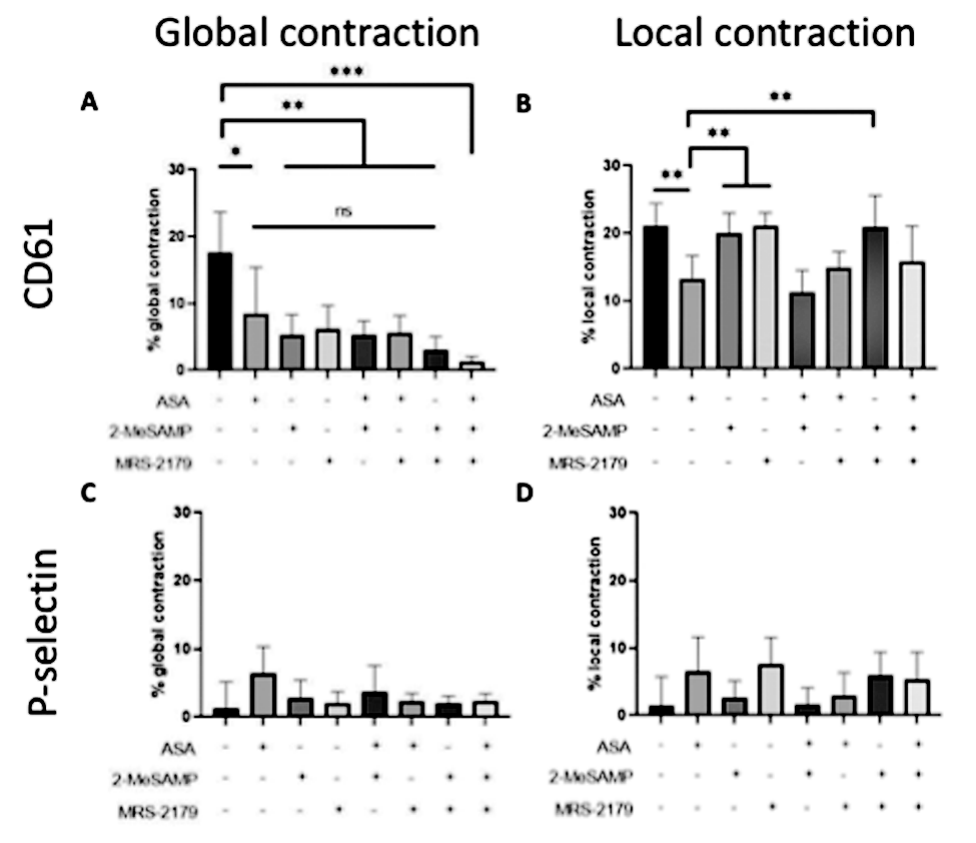
P-selectin + platelets have limited global and local contraction; ASA, 2-methylthio (Me)-SAMP, and MRS-2179 have a limiting effect on global platelet contraction, while only ASA limits local platelet contraction. Images from Fig. 1 were measured for global and local contraction. Global contraction measured the change in distance in clot length between 7.5 and 15 minutes images of a clot. Local contraction measured the change in percent area coverage of a clot between 7.5 and 15 minutes. Global contraction for platelets **(A)** , local contraction for platelets **(B)** , global contraction for P-selectin **(C)** , and local contraction for P-selectin **(D)** were compared among the various conditions. (N = 3 donors, *n* = 6 clots for each panel; * *p* < 0.05, ** *p* < 0.01, *** *p* < 0.001). ASA, acetylsalicylic acid.

Global and local contraction measurements were made based on images taken from experiments in Fig. 1 . Global contraction for platelet aggregates was measured as the percentage change in distance from the top edge of the clot to the bottom edge, between 7.5- and 15-minute images ( Fig. 3A ). Measurements for the control condition showed pronounced contraction. With individual ADP or TXA2 inhibitors added (ASA alone, 2-MeSAMP alone, or MRS-2179 alone), contraction was significantly reduced. Contraction was reduced even further when inhibitors were added in combination (ASA + 2-MeSAMP, ASA + MRS-2179, and 2-MeSAMP + MRS-2179), and decreased the most when all three inhibitors were present. This demonstrated an additive effect of ADP and/or TXA2 inhibitors on reducing global aggregate contraction.

Like global contraction, local contraction for platelet aggregates was pronounced under control conditions ( Fig. 3B ). However, unlike global contraction, local contraction for aggregates was affected differently by ADP and/or TXA2 inhibition. Conditions with 2-MeSAMP and MRS-2179 showed no consistent pattern in altering local aggregate contraction relative to control, suggesting ADP inhibition did not have much of an effect on local aggregate contraction. However, conditions with ASA consistently showed reduced local contraction, with significant differences. This suggested that TXA2 inhibition may play a role in limiting local aggregate contraction.

### P-Selectin + Platelets Have Limited Global and Local Contraction

In addition to CD61 + platelets, we also measured global ( Fig. 3C ) and local ( Fig. 3D ) contraction in P-selectin images. Global and local contractions for P-selectin in control were minimal relative to global and local aggregate (CD61 + ) contraction. This suggests that the P-selectin + core of the clot was less contractile relative to the P-selectin(-) shell region of the clot under control conditions. Additionally, ADP or TXA2 inhibition alone, or in combination, showed limited global and local contraction. Some ADP and/or TXA2 conditions showed marginally higher % contraction than control, but there did not appear to be a noticeably significant trend. Thus, the P-selectin + core of the clot was generally less contractile, and was not significantly affected by ADP or TXA2 inhibition.

### Platelet Fluorescence Correlates with Global Contraction

**Fig. 4:**
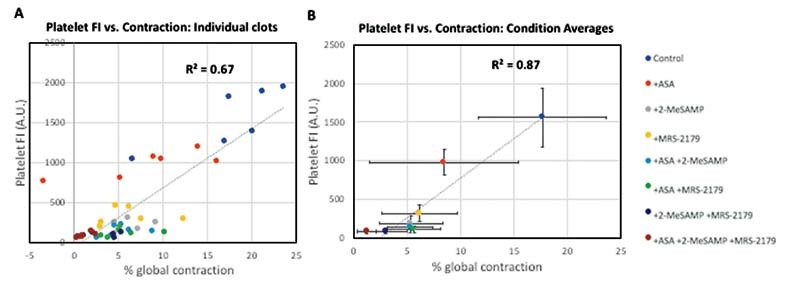
Platelet fluorescence correlates with global contraction. Final platelet fluorescence and % global contraction were graphed for individual clots **(A)** . Average fluorescence and contraction values for each condition were also graphed **(B)** . (N = 3 donors, *n* = 6 clots for B; AU = arbitrary units). FI, fluorescence intensity.

As observed in Fig. 2A , platelet FI was the greatest for control, while TXA2 inhibition with ASA noticeably reduced platelet FI. ADP inhibition (via either 2-MeSAMP or MRS-2179) significantly reduced platelet FI relative to control, and combinations of ADP and TXA2 inhibitors reduced platelet FI even further, suggesting ADP and TXA2 inhibition had an additive effect on reducing platelet deposition. In Fig. 3A , we saw contraction was greatest in control, and then significantly reduced when ADP and TXA2 inhibitors were present. ADP and TXA2 inhibition together showed the greatest reduction in global aggregate contraction, again suggesting ADP and TXA2 may have had an additive effect on reducing global aggregate contraction. Because of the potential additive effects of ADP and TXA2 inhibition on platelet deposition and global aggregate contraction, we wanted to see how platelet deposition and aggregate contraction correlated for each clot and for each condition. To do this, we compared the percentage of global aggregate (CD61 + ) contraction and platelet FI for each clot ( Fig. 4A ) and for the average of each clot within a given condition ( Fig. 4B ). With each clot graphed, there was a noticeable correlation ( *R* ^2^ = 0.67) between platelet fluorescence and global aggregate contraction ( Fig. 4A ). However, there was some deviation in individual clots; when the average of each condition was taken the correlation improves considerably ( *R* ^2^ = 0.87). This demonstrated an observable correlation between platelet FI and global aggregate (CD61 + ) contraction.

### Acetylsalicylic Acid Limits Local Aggregate Contraction, While 2-MeSAMP and MRS-2179 Have No Effect

**Fig. 5:**
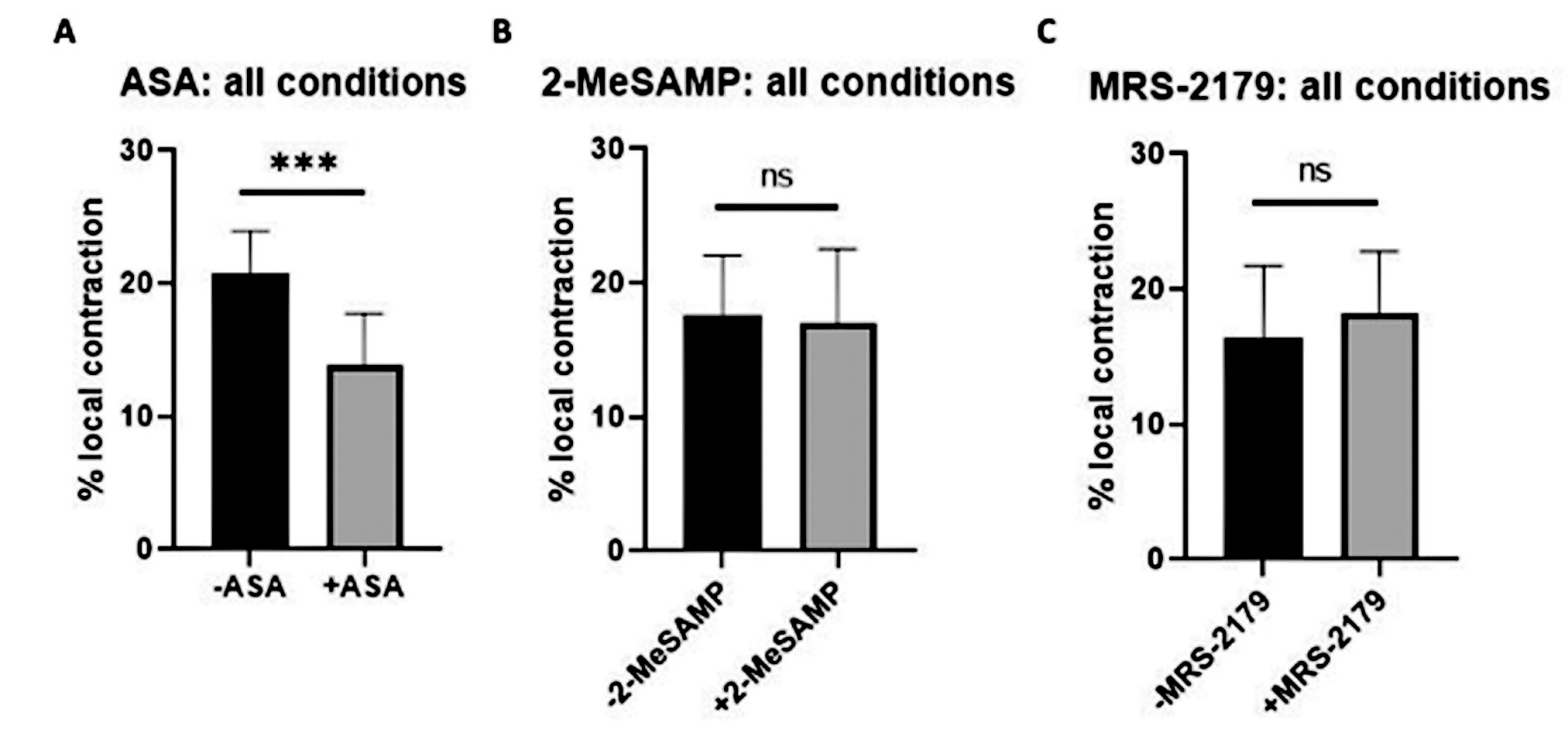
ASA limits local platelet contraction, while 2-methylthio (Me)-SAMP and MRS-2179 have no effect. We performed a meta-analysis of data from Fig. 3B by segregating data into different component conditions. Each of the 4 conditions with ASA and each of the 4 conditions without ASA were averaged and compared (A); the same was done for 2-methylthio (Me)-SAMP (B) and MRS-2179 (C). (N = 3 donors, *n* = 24 clots for each panel; * *p* < 0.05, ** *p* < 0.01, *** *p* < 0.001). ASA, acetylsalicylic acid.

In Fig. 3B , conditions that had ASA present tended to have reduced local aggregate contraction. To determine the significance that ASA had on limiting local aggregate contraction, we grouped all conditions that either had or did not have ASA ( Fig. 5A ), 2-MeSAMP ( Fig. 5B ), or MRS-2179 ( Fig. 5C ). When conditions were separated based on the presence or absence of 2-MeSAMP, there was no significant difference in local aggregate contraction ( Fig. 5B ). The same was true for MRS-2179 ( Fig. 5C ), suggesting ADP inhibition had no significant effect on local aggregate contraction. However, when conditions were separated based on the presence or absence of ASA, there was a significant difference ( *p* < 0.0001) in local aggregate contraction, with ASA conditions showing reduced contraction relative to conditions without ASA ( Fig. 5A ). This suggested that TXA2 inhibition may have had a limiting effect on local aggregate (CD61 + ) contraction.

## Discussion

Clot contraction is a key element of clot resolution and platelet contractility plays a significant role in contributing to contraction. Using our microfluidic device, we were able to evaluate clot contraction due from platelet contractility on a global and local basis in the presence of ADP and TXA2 inhibitors. To our knowledge, this is the first report evaluating platelet contractility in the presence of ADP and TXA2 inhibitors using microfluidics at a venous shear rate. We found that ASA, 2-MeSAMP, and MRS-2179 have an additive effect in limiting CD61 + platelet deposition but not with P-selectin + platelets. P-selectin + platelet aggregates had limited global and local contraction regardless of ADP or TXA2 inhibition. On the other hand, ADP and TXA2 inhibition seemed to have an additive effect on limiting global contraction of CD61 + platelet aggregates, while TXA2 inhibition, not ADP inhibition, limited local CD61 + aggregate contraction. Lastly, we found that platelet fluorescence correlates with global CD61 + aggregate contraction.

We have summarized these findings in Fig. 6 which illustrates our understanding of clot contraction in the presence or absence of ADP and TXA2 inhibition. When there is no ADP or TXA2 inhibition, we see significant CD61 + platelet deposition and P-selectin display at 7.5 minutes, and then after 7.5 minutes of flow cessation, CD61 + platelets contract locally which contributes to global contraction for CD61 + platelet aggregates. For P-selectin, there is limited local and global contractions. When ADP and TXA2 inhibitors are present, we see reduced CD61 + platelet deposition and reduced P-selectin + platelet deposition. We still generally see local CD61 + aggregate contraction (which is reduced by TXA2 inhibition), but because there is significantly less CD61 + platelets when ADP and TXA2 inhibitors are present, they are not able to affect global CD61 + aggregate contraction as much. Again, for P-selectin, there is limited local and global contraction.

**Fig.**
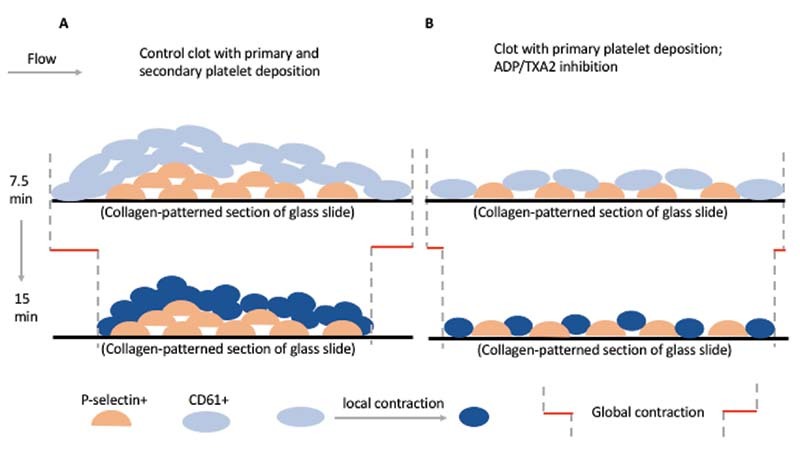
6 Schematic of clot development and contractile processes under control conditions **(A)** and with ADP/TXA2 inhibitors present **(B)** .

Our finding that P-selectin + platelets had limited contractility is in agreement with previous findings. 2 23 29 In the core/shell model of clot heterogeneity, P-selectin + platelets in the core are more closely packed, while P-selectin(-) platelets in the shell are loosely packed. 2 23 29 It would follow then that P-selectin + platelets would not be able to contract as much if they are closely packed, compared to CD61 + platelets in the shell region that are loosely connected and have greater ability to physically contract. We also observed that ADP inhibition and TXA2 inhibition each reduced CD61 + platelet deposition and P-Selectin + platelets in clots. However, we found that the combination of ADP and TXA2 inhibition further reduced CD61 + platelet deposition, while it did not further reduce P-selectin display. This may demonstrate that ADP and TXA2 inhibition may have a greater effect on P-selectin(-) shell platelets than on P-selectin + core platelets. Additionally, P-selectin + platelets are closer to collagen than shell platelets; ADP and TXA2 inhibition may no longer be effective at reducing P-selectin after a certain point, possibly due from collagen-induced P-selectin display.

We found that ASA limits local CD61 + aggregate contraction, while 2-MeSAMP and MRS-2179 did not. While the pharmacological mechanism for this difference should be studied further, it is likely not due from differences in clot morphology, since clots of varying degrees of platelet deposition are included in both +ASA and –ASA conditions ( Fig. 1A and B ), and there is a consistent decrease in percentage of local aggregate contraction in conditions with ASA compared to conditions without ASA ( Fig. 3B ). In terms of global CD61 + aggregate contraction, we found that ADP and TXA2 inhibition reduced aggregate contraction which is in agreement with previous findings. 6 We observed a trend where greater platelet deposition generally resulted in greater global aggregate contraction, which we confirmed in our data ( *R* ^2^ = 0.87; Fig. 5 ). While a greater number of platelets can physically limit the ability of a clot to contract, the more platelet-platelet interactions there are, the greater the contractile connectivity in the clot. In clots where ADP and TXA2 severely limited CD61 + platelet deposition, there is reduced platelet-platelet interactions, and as we saw in our results, ultimately less global aggregate contraction.

## Limitations and Strengths

It is important to note that this study is limited to evaluating platelet-driven contraction; in addition to platelets, leukocytes and RBCs can also be found in blood clots. It has been shown that monocytes can aid platelet-driven contraction. 30 In the case of ischemic stroke patients, thrombi with greater leukocyte composition were associated with increased fatality. 31 In contrast to leukocytes, RBCs impair clot contraction. 3 RBCs contracting within a clot have a polyhedron shape, forming a compact array of polyhedral structures. 8 32 In patients with sickle cell disease, impaired clot contraction is due from decreased RBC deformability. 33 Future studies should focus further on evaluating the roles of leukocytes and RBCs on global and local aggregate contraction.

Fibrinogen plays an important role in platelet aggregation. Fibrinogen binds platelets via integrin aIIbB3 which allows for platelet-platelet interactions. However, fibrinogen has been shown to limit clot contraction. 3 It has also been shown that ischemic stroke patients had higher fibrinogen levels compared to healthy subjects. 14 While fibrinogen was not extensively investigated in this study, it should be studied further for its limiting effect on clot contraction at higher concentrations. 3

Impaired clot contraction has been implicated as a complicating factor in a number of conditions. Clinical correlations based on patient data suggest that impaired clot contraction may be a risk factor for ischemic stroke. 14 Reduced clot contraction has also been seen in patients with venous thromboembolism. 12 13 Hyperhomocysteinemia, or increased homocysteine levels, causes excessive platelet activation and impairs contraction, making clots larger and more obstructive. 34 Reduced clot contraction in blood of systemic lupus erythematous patients impairs blood flow and could be a risk factor for increased thrombosis. 35 In sickle cell patients, clots have impaired contraction due to pathological stiffening of RBCs in blood clots. 33 Reduced clot contraction is a pathological risk factor for and is correlated with a number of prothrombotic conditions. This study and future studies focused on better understanding clot contraction will help in our ability to better mitigate and resolve these types of prothrombotic conditions.

## Conclusion

These results demonstrate a necessary balance in platelet deposition and aggregate contraction. For hemostasis to be properly maintained, hyperactivation of platelets must be limited to prevent thrombosis or embolization, but there also must be sufficient platelet deposition to allow for a clot to form and subsequently contract. ADP and TXA2 inhibitors seek to alter this balance by preventing thrombosis but too much inhibition can result in bleeding. Part of the reason for this potential bleeding, from our results here, could be due from ADP and TXA2 inhibitors' ability to limit clot contraction and clot resolution. Understanding the fundamental mechanisms behind how these inhibitors affect clot contraction has potentially significant clinical benefits in deciding how to treat patients who may require antiplatelet agents.

## Notes

**What Is Known about This Topic?**

- Clot contraction is an important part of hemostasis to ensure clot stability and ultimately clot resolution.
- Contraction is a complex process that has been extensively studied under entirely static conditions.
- ADP and thromboxane (TX) A2 inhibitors have been previously shown to limit platelet contractility.

**What Does This Paper Add?**

- We developed two distinct metrics for evaluating clot contraction in physiologically relevant clot morphologies.
- We found that ADP and TXA2 inhibited global aggregate contraction. TXA2 inhibition limited local aggregate contraction while ADP inhibition did not.
- P-selectin + platelets were much less contractile (global and local) than CD61 + platelets.
