# Supplementary material for: ADP and Thromboxane Inhibitors Both Reduce Global Contraction of Clot Length, While Thromboxane Inhibition Attenuates Internal Aggregate Contraction
Source: TH Open. 2022 Jun 13;6(2):e135–43. doi: 10.1055/a-1832-9293 (PMC9192180; doi:10.1055/a-1832-9293)
Supplement: Supplementary file 1 — Supplementary Material [file 10-1055-a-1832-9293-s220004.pdf]

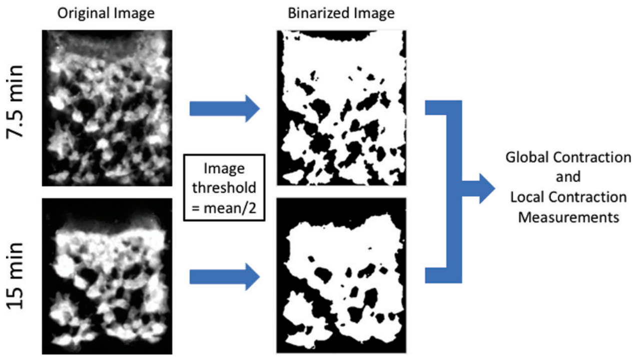

Supplementary Fig. 1 Explanation of image thresholding process. Images (for both platelets and P-selectin fluorescence) were made binary in Matlab for contraction analysis. The threshold value for binary images was 1/2 of the mean fluorescence of each image. This threshold value was used consistently across all images. Once binarized, clots were analyzed for global and local contraction.

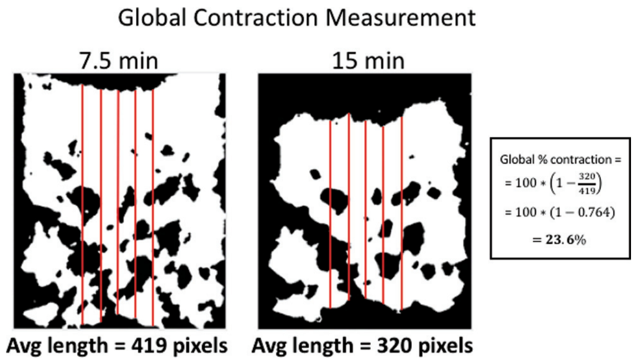

Supplementary Fig. 2 Example global contraction measurement. Once images were binary, measurements were made at 5 different points in the x direction of each clot: at the central pixel, ± 25 pixels, and ± 50 pixels. These measurements were then averaged and used to calculate the global percentage contraction for the clot.

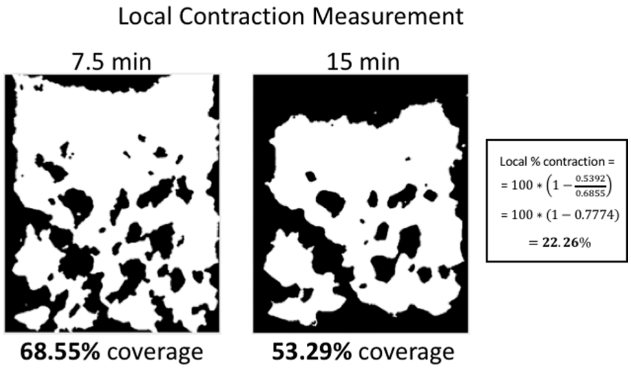

Supplementary Fig. 3 Example local contraction measurement. Once images were binary, the percentage of the clot area covered by platelets (white) was calculated in Matlab for each image of a clot (at 7.5 and 15 min). The percentage area coverage for each image was used to calculate the local percentage contraction for the clot.

 © 2022. The Author(s).
